# Case Report: Persistent Hypogammaglobulinemia More Than 10 Years After Rituximab Given Post-HSCT

**DOI:** 10.3389/fimmu.2021.773853

**Published:** 2021-12-22

**Authors:** Fanny Luterbacher, Fanette Bernard, Frédéric Baleydier, Emmanuelle Ranza, Peter Jandus, Geraldine Blanchard-Rohner

**Affiliations:** ^1^ The Children’s Hospital, Faculty of Medicine, Geneva University Hospitals, Geneva, Switzerland; ^2^ Pediatric Hematology/Oncology Unit, Faculty of Medicine, Geneva University Hospitals, Geneva, Switzerland; ^3^ Genetic Medicine Division, Geneva University Hospitals, Geneva, Switzerland; ^4^ Medigenome, Swiss Institute of Genomic Medicine, Geneva, Switzerland; ^5^ Immunology and Allergology Division, Faculty of Medicine, Geneva University Hospitals, Geneva, Switzerland; ^6^ Pediatric Immunology and Vaccinology Unit, General Pediatrics Division, Department for Women, Children, and Teenagers, Geneva University Hospitals and University of Geneva, Geneva, Switzerland

**Keywords:** rituximab, hypogammaglobulinemia, HSCT, children, immunological workup

## Abstract

Rituximab (RTX) is an anti-CD20 monoclonal antibody that targets B cells—from the immature pre-B-cell stage in the bone marrow to mature circulating B cells—while preserving stem cells and plasma cells. It is used to treat autoimmune diseases, hematological malignancies, or complications after hematopoietic stem cell transplantation (HSCT). Its safety profile is acceptable; however, a subset of patients can develop persistent hypogammaglobulinemia and associated severe complications, especially in pediatric populations. We report the unrelated cases of two young men aged 17 and 22, presenting with persistent hypogammaglobulinemia more than 7 and 10 years after treatment with RTX, respectively, and administered after HSCT for hemolytic anemia and Epstein–Barr virus reactivation, respectively. Both patients’ immunological workups showed low levels of total immunoglobulin, vaccine antibodies, and class switched-memory B cells but an increase in naive B cells, which can also be observed in primary immunodeficiencies such as those making up common variable immunodeficiency. Whole exome sequencing for one of the patients failed to detect a pathogenic variant causing a Mendelian immunological disorder. Annual assessments involving interruption of immunoglobulin replacement therapy each summer failed to demonstrate the recovery of endogenous immunoglobulin production or normal numbers of class switched-memory B cells 7 and 10 years after the patients’ respective treatments with RTX. Although the factors that may lead to prolonged hypogammaglobulinemia after rituximab treatment (if necessary) remain unclear, a comprehensive immunological workup before treatment and long-term follow-up are mandatory to assess long-term complications, especially in children.

## Introduction

Rituximab (RTX) is an anti-CD20 chimeric monoclonal antibody used to treat numerous autoimmune diseases [e.g., autoimmune hemolytic anemia (AIHA) and thrombocytopenia], neurological immunological diseases (e.g., multiple sclerosis and small fiber neuropathy), various vasculitis and connective tissue diseases, and some hematological B-cell malignancies (1, 2). However, previous reports have shown that RTX may be responsible for severe hypogammaglobulinemia (3–6). It reduces numbers of B lymphocytes *via* the direct toxic effects of Fc receptor, gamma-mediated, antibody-dependent cytotoxicity and phagocytosis, complement-mediated cell lysis, growth arrest, and B-cell apoptosis (7, 8). RTX very often induces hypogammaglobulinemia, especially following hematopoietic stem cell transplantation (HSCT) (3), but this is generally transient and recovers slowly after an average period of 12 months (2, 9). However, several cases of prolonged hypogammaglobulinemia have been described previously (3, 4, 10–12). Here, we describe the cases of two young men, aged 17 and 22 years old, presenting with prolonged hypogammaglobulinemia persisting more than 7 and 10 years, respectively, and receiving post-HSCT RTX treatment for AIHA and Epstein–Barr virus (EBV) reactivation, again, respectively. They underwent close and regular immunological follow-up for more than 10 years.

## Case Presentations

Patient 1 was a 22-year-old young adult who had had a fulminant hepatic failure of unknown origin aged 10 years old, followed by a very severe aplastic anemia (vSAA) in February 2009. Almost simultaneously, he developed acute fibrinous and organizing pneumonia in March 2009. In August 2009, 5 months after the initial diagnosis of a vSAA, HSCT was performed using cells from an unrelated 10/10 matched donor after a conditioning regimen including anti-thymocyte globulin (5 mg/kg), fludarabine (150 mg/m^2^), cyclophosphamide (5.8 mg/m^2^), and cyclosporine as prophylaxis for acute graft versus host disease (GVHD). The transplant was successful, with 100% donor whole blood chimerism, and remained so from October 2009 until now. After engraftment, he nevertheless developed cutaneous grade 1 acute GVHD (treated with corticosteroids), mild renal thrombotic microangiopathy, and then autoimmune hemolytic anemia (AHAI), which was treated using corticosteroids and a 4-week course (375 mg/m^2^ per week) of IV RTX in July 2011. Lymphocyte immunophenotyping was normal (B cells, T cells, and NK cells) before RTX administration. T-cell proliferation studies of mitogens and specific antigens were normal. By March 2012, the AHAI had resolved. Anti-infectious prophylaxis and immunoglobulin replacement therapy (IRT) were then stopped. Before HSCT, IgG, IgA, and IgM levels were all normal. In June 2012, IgG trough levels were 2 g/L, gradually dropping to 0.67 g/L in February 2013. Clinically, the patient presented with no severe infections.

**Figure 1:**
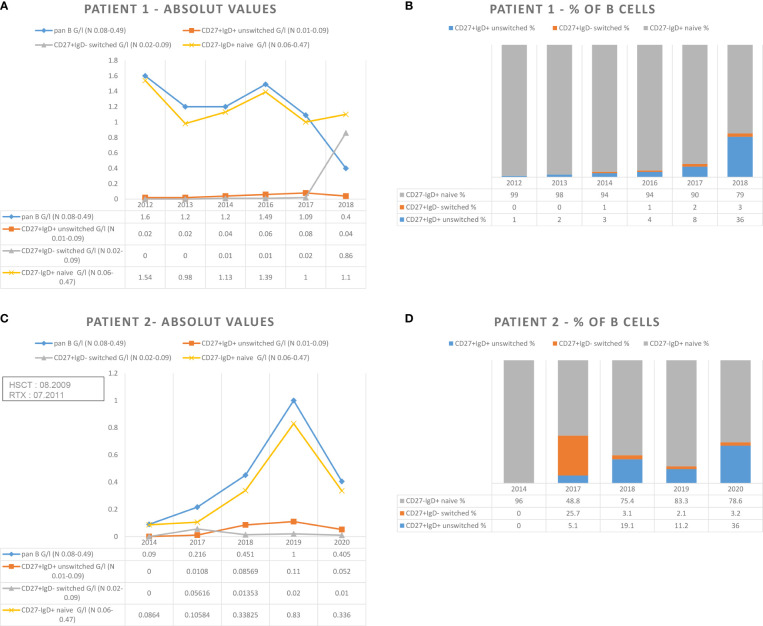
Absolute number **(A, C)** and percentage **(B, D)** of naïve and unswitched- and switched-memory B cells in patient 1 and 2.

A thorough immunology workup was done in November 2012, showing IgG at 0.8 g/L, IgM at 0.06 g/L, and undetectable IgA. Vaccine antibodies were not protective for diphtheria, tetanus, Hib, or any tested pneumococcal serotypes (14, 19, 23F, 9N, 11A, and 17F), but they remained positive for varicella and measles (see **Table 1**). Lymphocyte immunophenotyping showed a normal B-cell count (420 cells/µl) but fewer unswitched-memory B cells (1%; when normal = 4%–23%) and switched-memory B cells (0%; when normal = 5%–18%) and more naive B cells (99%; when normal = 60%–89%) (**Figure 1**). The patient was unresponsive to diphtheria, tetanus, pertussis, and poliomyelitis vaccination (Infanrix^®^) in December 2012 (**Table 1**).

**Table 1:** Concentrations of immunoglobulins and vaccine antibodies in patients 1 and 2.

Patient 1													
	2009	2010	2011	2012	2013	2014	2015	2016	2017	2018	2019	2020	2021
Weeks of IVIG interruption	No Ivig	No Ivig	No Ivig	No Ivig	3	8	10.5	14	16	16	6.5	8.5	8
Immunoglobulins													
IgG g/L (N7–16)	8.19	3.95	3.49	0.82	0.67	2.61	3.62	3.47	3.15	3.35	6.87	3.82	4.29
IgA g/L (N0.7–4)	2.31	0.3	0.36	<0.06	<0.06	<0.06	<0.06	<0.06	<0.06	<0.06	<0.06	<0.06	<0.06
IgM g/L (N0.4–2.3)	0.4	0.18	0.26	0.05	0.05	0.32	0.9	0.35	1.65	1.39	1.67	0.9	0.73
Vaccinal antibodies													
Tetanus, IgG ELISA UI/L (N > 100)	1,966	1,762	1,408	<100	767	–	865	833	468	–	–	–	2,382
Diphteria, IgG ELISA UI/L (N > 100)	275	481	475	<100	162	–	222	206	145	–	–	–	745
Varicella, IgG ELISA UI/L (N > 50)	–	–	–	163	–	–	–	–	295	–	–	–	>2,000
Measles, IgG ELISA qn UI/L (N50–150)	–	–	–	–	–	–	–	–	157	–	–	–	>1,000
Hemophilus b, IgG ELISA mg/l (N > 0.15)	–	2.13	1.72	<0.15	1.34	–	–	–	–	–	–	–	4.17
Pneumococcus 14, IgG par ELISA mg/L (N > 0.3)	1	105	2.3	<0.3	1	–	1.3	1.5	1.5	2.5	–	–	>5
Pneumococcus 19, IgG par ELISA mg/L (N > 0.3)	10	404	1.8	0.4	0.9	–	>5	1.2	4	1.5	–	–	3.7
Pneumococcus 23F, IgG par ELISA mg/L (N > 0.3)	0.6	0.5	0.6	<0.3	0.6	–	>5	0.9	2.9	0.8	–	–	4.2
Pneumococcus 9N, IgG par ELISA mg/L (N > 0.3)	–	–	–	<0.3	–	–	–	0.4	1.1	0.7	–	–	–
Pneumococcus 11A, IgG par ELISA mg/L (N > 0.3)	–	–	–	<0.3	–	–	–	0.6	0.5	1	–	–	–
Pneumococcus 17F, IgG par ELISA mg/L (N > 0.3)	–	–	–	<0.3	–	–	–	0.5	0.4	0.8	–	–	–
Patient 2													
						2014	2015	2016	2017	2018	2019	2020	2021
Weeks of IVIG interruption						No Ivig	4	6	12	10	8	8	2
Immunoglobulins													
IgG g/L (N7–16)						6.37	3.42	3.63	2.29	4.83	3.25	2.88	8.46
IgA g/L (N0.7–4)						<0.06	<0.06	<0.06	<0.07	<0.06	<0.06	<0.06	<0.06
IgM g/L (N0.4–2.3)						<0.04	1.71	0.77	1.11	1.38	1.33	2.32	2.14
Vaccinal antibodies													1
Tetanus, IgG ELISA UI/L (N > 100)						395	–	–	–	240	–	136	1,075
Diphteria, IgG ELISA UI/l (N > 100)						<100	–	–	–	<100	–	<100	407
Varicella, IgG ELISA UI/L (N > 50)						>2,000	–	–	–	–	–	168	1,866
Measles, IgG ELISA qn UI/L (N50–150)						191	–	–	–	–	–	<100	391
Hemophilus b, IgG ELISA mg/L (N > 0.15)						–	–	–	–	0.32	–	7.66	6.04
Pneumococcus 14, IgG par ELISA mg/L (N > 0.3)						0.9	–	–	–	–	–	<0.3	2.1
Pneumococcus 19, IgG par ELISA mg/L (N > 0.3)						1.8	–	–	–	0.5	–	<0.3	–
Pneumococcus 23F, IgG par ELISA mg/L (N > 0.3)						<0.3	–	–	–	<0.3	–	<0.3	0.7
Pneumococcus 9N, IgG par ELISA mg/L (N > 0.3)						<0.3	–	–	–	–	–	<0.3	–
Pneumococcus 11A, IgG par ELISA mg/L (N > 0.3)						<0.3	–	–	–	–	–	<0.3	–
Pneumococcus 17F, IgG par ELISA mg/L (N > 0.3)						<0.3	–	–	–	–	–	<0.3	–

IRT with Privigen^®^ (1 g/kg/month) was started in March 2013 and interrupted during the summer to evaluate possible immune reconstitution. Every attempt to discontinue treatment showed a recurrence of hypogammaglobulinemia and respiratory tract infections. Regular monitoring of lymphocyte subpopulations showed persistent low unswitched- and switched-memory B cells, whereas the total number of B cells increased temporarily. IgA levels remained undetectable, and IgM levels increased over time, reaching normal values. Similar signs can be observed in primary immunodeficiencies, such as common variable immunodeficiency (CVID).

Whole-exome sequencing using a targeted bioinformatics analysis of a panel of 478 genes involved in Mendelian immunological disorders was performed on DNA extracted from a buccal smear, but this was unable to identify any pathogenic or likely pathogenic variants causative of hypogammaglobulinemia. The donor was not known to have a primary immunodeficiency.

**Figure 2:**
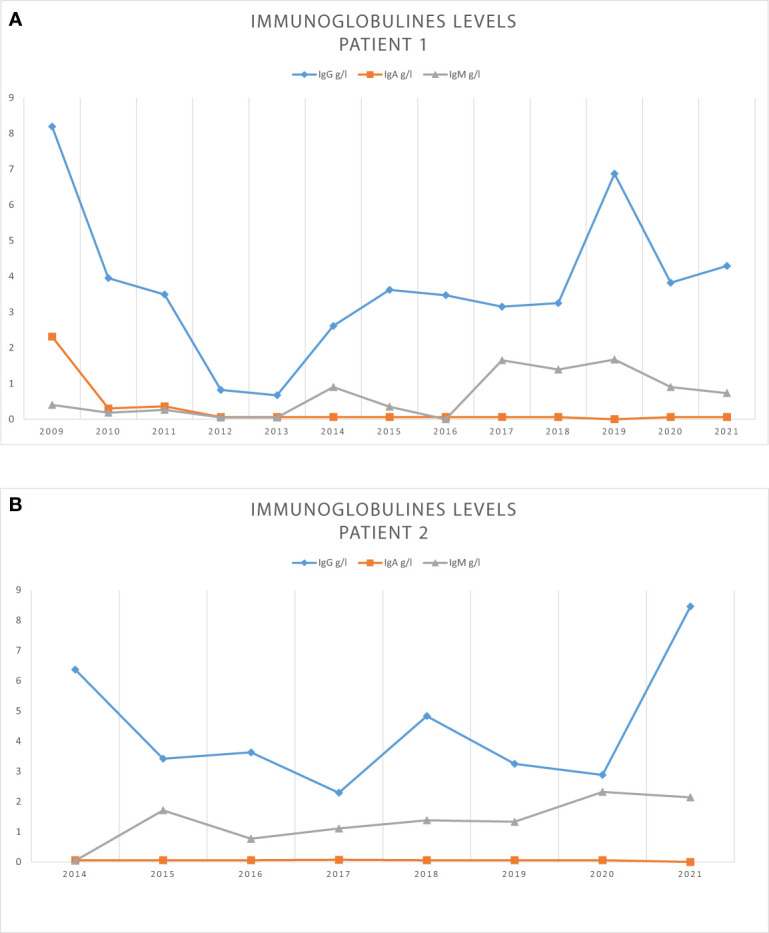
Immunoglobulies levels during folow up for patient 1 **(A)** and patient 2 **(B)**.

The second patient was a 17-year-old male adolescent, in good general health except for recurrent bronchitis and pharyngitis in infancy that necessitated an amygdalotomy at 2 years old. In March 2010, at 11 years old, he presented with a high-risk acute pre-B-lymphoblastic leukemia [hyperleukocytosis, central nervous system (CNS) status 2b, translocation t(9;12) on cytogenetics screening]. He was initially treated using chemotherapy according to Children’s Oncology Group (COG) protocol AALL0232 until July 2011. He also received craniospinal radiotherapy of 12 Gy in total. Two months after the end of treatment, he had an early relapse, with acute lymphoblastic leukemia involving both his marrow and left testis. He was treated according to COG protocol AALL0433 in October 2013. Response to treatment in the bone marrow was delayed, with 5%–10% of blasts persisting in the marrow after reinduction 2. Nevertheless, cytological remission was obtained at the end of reinduction 3, with a positive minimal residual disease of 0.03%. However, there was an absence of testis remission after three cycles of induction. The patient received a fourth course of chemotherapy in January 2014 and underwent a left orchidectomy. He suffered a medullary relapse just before the transplant, which was treated with clofarabine, cyclophosphamide, and etoposide, and then received an unrelated HSCT with 10/10 human leukocyte antigen compatibility in March 2014. Conditioning was performed using total body irradiation (12 Gy in six fractions preceded by a testicular boost of 6 Gy in three fractions), etoposide (60 mg/kg), and anti-thymocyte globulin (25 mg/kg). The transplant involved 100% donor whole-blood chimerism and was successful from April 2014 until now. The post-transplant course of treatment was complicated by EBV reactivation, which was treated using RTX in May 2014 (4 doses 375 mg/m^2^ IV weekly). The patient received no dosages of IgG, IgA, or IgM and underwent no lymphocyte immunophenotyping before HSCT. By the end of October 2014, the pan B cells were normal, but IgG trough levels were approximately 6 g/L, with very low IgA and IgM, despite continued IRT (Privigen^®^) at 0.4 g/kg monthly (see **Figure 2**). In February 2015, extended lymphocyte immunophenotyping showed normal pan B-cell levels but lower levels of switched- and unswitched-memory B cells. He stopped IRT between July 2016 and October 2016, with IgG levels dropping to 3.6 g/L in October 2016, and then remained under IRT (Privigen^®^) at 0.4 g/kg per month until June 2017. At that time, he had an IRT interruption to assess any possible immune reconstitution, but IgG levels dropped to 2.29 g/L in October 2017. Lymphocyte immunophenotyping showed normal total B cells and switched- and unswitched-memory B cells (see **Table 1**). IRT was restarted. A new IRT interruption took place in April 2019; however, after 8 weeks, IgG levels had dropped to 3.25 g/L. In June 2019, total B cells were normal, as were the unswitched-memory B cells. However, the switched-memory B cells were in the lower normal ranges. IRT was therefore resumed. In September 2019, IRT was again interrupted temporarily for 12 weeks and residual IgG levels dropped to 4.3 g/L. As a result, clinicians decided to recommence IRT (Privigen^®^) at a dosage of 0.4 g/kg at 8-week intervals, which maintained IgG trough levels at 5 g/L. In July 2020, after a 10-week IRT interruption, IgG levels were at 6 g/L. In all previous years, IgA had been undetectable, while IgM was normal (see **Figure 2**). Vaccine antibodies were low for all vaccine antigens in July 2020. Extended lymphocyte immunophenotyping showed a slight decrease in switched-memory B cells, whereas unswitched-memory B cells were normal and naive B cells had increased. Booster doses of various vaccines (PCV13 and Infanrix-hexavalent^®^) were administered in July 2020. In August 2020, he was unresponsive to PCV13 and Infanrix-hexavalent^®^. The IgG levels dropped to 2.9 g/L in December 2020, and vaccine antibodies had dropped, too (see **Table 1**). Weekly subcutaneous IRT was initiated using Cuvitru^®^ 10 g (0.2 g/kg) in the first week, followed by 5 g (0.1 g/kg) weekly in the following weeks. Lymphocyte immunophenotyping showed normal amounts of B, T, and NK cells but fewer switched-memory B cells (**Table 1**). T-cell proliferation studies of mitogen and specific antigens were normal.

## Discussion

RTX is frequently used to treat HSCT complications, such as EBV reactivation, hemolytic anemia, and EBV-induced post-transplant lymphoproliferative disease (13). It has been reported that post-RTX treatment hypogammaglobulinemia is more frequent in patients with malignant diseases (14). In contrast, in a randomized controlled trial of acute rejection after kidney transplantation, in which 20 children were treated with RTX, the mean time to peripheral CD19 cell recovery was approximately 12 months (15). Suggested possible predisposing factors for prolonged post-RTX treatment hypogammaglobulinemia have been the number of RTX doses, baseline immunoglobulin levels, and the combination of RTX with mycophenolate or purine analogues (10, 14, 16, 17). Persistent hypogammaglobulinemia (>2 years) was observed in 32% of a patient cohort of 53 children from 16 European centers who received RTX for childhood cytopenia (5). The following risk factors were identified: young age at presentation, better treatment response, and a diagnosis of autoimmune hemolytic anemia or Evans syndrome (5). In addition, children with persistent hypogammaglobulinemia were more likely to have an underlying primary immunodeficiency (PID), such as CVID, other primary antibody deficiencies, or an activated PI3K-delta syndrome (5). Lum et al. reported on a cohort of patients, transplanted because of PID, who developed post-HSCT autoimmune cytopenia. Of those treated with RTX, 29% (5/17) developed a persistent low B-lymphocyte count and remained on IRT (10). These five patients with persisting hypogammaglobulinemia also had undetectable levels of IgM. In contrast, in our two patients, total B-cell counts were normal, although the switched-memory B cells remained low and IgM levels were normal, suggesting that there was no correlation between total B cells and IgM levels and prolonged hypogammaglobulinemia. Similarly, another retrospective cohort study of pediatric patients, treated with RTX for various reasons other than complications relating to HSCT, also showed that an underlying PID was relatively common in children developing hypogammaglobulinemia after treatment with RTX, thus highlighting the importance of immunological assessment (6). Low total IgG levels, vaccine antibodies, and switched-memory B cells have also been seen in patients with CVID (18). Alternatively, in certain individuals, this immunodeficiency might have been present before allogeneic HSCT and unmasked by RTX treatment in the context of a partial chimerism of the B-cell population. However, our patients had displayed total donor chimerism until now, and we were not informed of any PID present in the HSCT donors. Our first patient displayed several features of immune dysregulation, with numerous autoimmune complications (autoimmune hepatitis, aplastic anemia, and post-HSCT autoimmune hemolytic anemia and autoimmune neutropenia), suggesting a PID. Exome sequencing with targeted bioinformatics analysis was unable to identify a pathogenic or likely pathogenic variant in the genes involved in known Mendelian immunological conditions.

In another study of 14 patients treated with autologous peripheral blood stem cell transplantation and adjuvant therapy using RTX for high-risk CD20+ lymphoma, 43% of patients were found to have persistent hypogammaglobulinemia after a median follow-up of 32 months (3). Phenotypic analysis revealed that these patients had already achieved B-cell recovery, but with a severe delay in memory B-cell recovery, especially in the switched populations; naive B cells had normalized, however. Interestingly, they found that RTX decreased not only the quantity of B cells but also their quality, resulting in a reduced B-cell repertoire similar to that seen in CVID patients (3). Likewise, naive B-cell levels in our two patients were normal, but there were fewer switched-memory B cells. Another retrospective study of six children after HSCT showed that the mean recovery time of B lymphocytes was 353 ± 142 days in the children treated with RTX compared with 122 ± 45 days in the controls (19). Thus, RTX likely plays an important role in the reconstitution of the B-cell lineage after HSCT.

Overall, the pathophysiology of prolonged hypogammaglobulinemia after RTX treatment and the factors predisposing this condition remain unclear. However, our two cases suggested that RTX treatment after HSCT may lead to persistent and prolonged hypogammaglobulinemia with an increased risk of severe infections (6). Treating physicians should be aware not only of the desired short- and medium-term effects but also of the possible long-term complications of RTX treatment. An immunological workup is mandatory before starting this treatment because it has been observed that low IgG levels and B-cell counts before RTX treatment were among the factors affecting B-cell reconstitution after it (10). Our observations led us to conclude that despite the normalization of total B-cell counts and IgM levels, hypogammaglobulinemia persisted after RTX treatment. Although there were sometimes increases in total numbers of B cells and memory B-cell subsets, these were only transitory and not associated with the normalization of IgG levels. In addition, lower IgG levels were associated with lower B-cell function, as booster vaccinations were not followed by increases in vaccine antibodies, suggesting that these patients remained at risk of infections and should receive long-term IRT. However, close immunological monitoring during a summer break from IRT may help clinicians to assess the recovery of endogenous immunoglobulin production after a certain time. Further research is needed to demonstrate the factors and pathophysiology that predispose patients to developing this complication and to understand whether it can persist throughout life or eventually regresses over time.

## Data Availability Statement

The original contributions presented in the study are included in the article/supplementary material. Further inquiries can be directed to the corresponding author.

## Ethics Statement

Ethical review and approval was not required for the study on human participants in accordance with the local legislation and institutional requirements. The patients/participants provided their written informed consent to participate in this study. Written informed consent was obtained from the individual(s) for the publication of any potentially identifiable images or data included in this article.

## Author Contributions

FL wrote the manuscript. FL, FBe, FBa, ER, PJ, and GB-R contributed to patients’ care, data collection, and interpretation. GB-R coordinated the study, was involved in the care of the patients, and wrote the manuscript. All authors contributed to the article and approved the submitted version.

## Conflict of Interest

The authors declare that the research was conducted in the absence of any commercial or financial relationships that could be construed as a potential conflict of interest.

## Publisher’s Note

All claims expressed in this article are solely those of the authors and do not necessarily represent those of their affiliated organizations, or those of the publisher, the editors and the reviewers. Any product that may be evaluated in this article, or claim that may be made by its manufacturer, is not guaranteed or endorsed by the publisher.
